# Associations Among Monoamine Neurotransmitter Pathways, Personality Traits, and Major Depressive Disorder

**DOI:** 10.3389/fpsyt.2020.00381

**Published:** 2020-05-13

**Authors:** Xiaojun Shao, Gang Zhu

**Affiliations:** ^1^ Department of Psychiatry, The First Affiliated Hospital of China Medical University, Shenyang, China; ^2^ Central Laboratory, The First Affiliated Hospital of China Medical University, Shenyang, China

**Keywords:** personality traits, mood disorder, major depressive disorder, monoamine neurotransmitters, mechanism

## Abstract

Major depressive disorder (MDD) is a complex psychiatric disease requiring multidisciplinary approaches to identify specific risk factors and establish more efficacious treatment strategies. Although the etiology and pathophysiology of MDD are not clear until these days, it is acknowledged that they are almost certainly multifactorial and comprehensive. Monoamine neurotransmitter system dysfunction and specific personality traits are independent risk factors for depression and suicide. These factors also demonstrate complex interactions that influence MDD pathogenesis and symptom expression. In this review, we assess these relationships with the aim of providing a reference for the development of precision medicine.

## Introduction

Major depressive disorder (MDD) is the most prevalent mood disorder and the most common disabling psychiatric disease across the globe. In the United States, the lifetime prevalence of MDD is 20.6% (1), and the associated healthcare and economic burdens are surpassed only by cardiomyopathy (2). The most clinically significant symptom of MDD is suicidality (3, 4). Over the years, MDD has been explained in genetic, biological, psychosocial, personality and other terms. No definite explanation accounts for the mechanism of MDD, however. Reducing the morbidity and mortality associated with MDD requires a more complete understanding of disease pathophysiology. Evidence accrued over many decades strongly implicates dysregulation of monoamine neurotransmitter systems in MDD development. Further, there is compelling evidence that MDD risk is strongly associated with certain personality traits. In this review, we expound the underlying relationships among monoamine neurotransmitter systems, personality traits, and MDD.

A biological basis for MDD risk is strongly supported by genetic studies demonstrating moderate heritability (ranging from about 37% and 45%) (5–9). Thus, gene–environment interactions are likely crucial to disease etiology, such as stressful life events (10, 11), childhood maltreatment (including emotional abuse, sexual abuse, emotional neglect, and physical neglect) (12, 13), and in fact these interactions result in an underestimation of the overall genetic influence (14). Kendler et al. reported a genetic correlation for liability to major depression of 0.63 in both males and females (9), and a similar estimate was reported in a population-based twin study (0.55) (15), consistent with several earlier studies suggesting that genetic risk factors are not sex-specific (16–19). However, the largest-sample twin study reported greater heritability in females (0.49, 95%CI = 0.31─0.56 vs. 0.41, 95%CI = 0.21─0.49), as well as 0.36 (95%CI = 0.31─0.38) in full siblings and 0.51 (95%CI = 0.51─0.53) in half-siblings (20). Several other studies have found a similarly elevated genetic propensity in females (9, 21, 22). These observed differences in MDD heritability between males and females are particularly interesting because recent neuroimaging and molecular genetic studies have also shown potential biological differences in MDD etiology between men and women. Edvardsen et al. reported a higher monozygotic/dizygotic ratio among male twins compared to female twins (8). Alternatively, a sex-limitation model suggested that the same genes influence MDD in males and females (19), although others have found that different genes impacted depressive illness (23). Thus, there is still no consensus on sex differences in the genetics of MDD.

## Monoamine Neurotransmitters and MDD

Multiple studies have implicated the monoamine neurotransmitters 5-hydroxytryptamine (5-HT or serotonin), dopamine (DA), and norepinephrine (NE) as the primary contributors to MDD etiology. In the mammalian central nervous system (CNS), the major sources of the three monoamines are the raphe nuclei (24), substantia nigra and ventral tegmentum area (VTA) (25), and locus coeruleus, respectively.

Raphe serotonergic neurons project to the caudate, putamen, pallidus, amygdala, limbic forebrain, and neocortex, where 5-HT signaling contributes to motivation, emotion stress processing (26), and regulation of other limbic functions (27). Acute depletion of the 5-HT precursor tryptophan (acute tryptophan depletion, ATD) markedly influences affective experience and emotional regulation in subjects with a family history of MDD (28). Challis et al. reported sensitization of inhibitory GABAergic neurons within the dorsal raphe nuclei and concomitant inhibition of serotonergic activity following social defeat in mice (29). Collectively, human and animal studies of tryptophan depletion (30) and associated serotonergic signaling deficiency strongly implicate 5-HT in mood regulation and MDD pathogenesis. Such insufficient 5-HT signaling may result from both reduced release and lower postsynaptic sensitivity as MDD patients demonstrate both decreased plasma and platelet levels of 5-HT, as well as blunted prefrontal cortical responses to 5-HT (31). Barton et al. reported elevated brain serotonin turnover before antidepressant therapy and markedly reduced turnover after antidepressant therapy and condition improvement, suggesting brain serotonin turnover as a potential biomarker for MDD (32). Further, a recent positron emission tomography (PET) study found reduced binding potential of the 5-HT_1A_ receptor subtype in MDD patients relative to controls, and the authors suggested that lower 5-HT_1A_ activity may result in “decreased engagement of the cognitive control network and impaired resolution of interfering cognitive stimuli” (33). Also consistent with a major contribution of 5-HT signaling dysfunction to MDD, elevated brain turnover of 5-HT is strongly influenced by 5-HT transporter (5-HTT) genotype (32), which in turn is associated with MDD risk. The urine serotonin/dopamine ratio may also be a useful diagnostic indicator for patients with MDD (34). Alternatively, selective serotonergic reuptake inhibitors (SSRIs) like fluoxetine, fluvoxamine, paroxetine, sertraline, and citalopram can enhance brain serotonin levels and are considered the first-line therapies for MDD patients based on demonstrated efficacy in the majority of placebo-controlled clinical studies (35). Growing evidence supports the hypothesis that epigenetic mechanisms, such as DNA methylation, play an important role in psychiatric diseases (36) such as MDD and personality disorders (37, 38), where epigenetic factors bridge the environmental and genetic mechanisms. A multitude of reports have considered the DNA methylation of the serotonin transporter gene (SLC6A4), located on chromosome 17 (39), as the major research target in investigation and evaluation in depression (**Table 1**). In summary, 5-HT is the biogenic amine most strongly associated with depression, as evidenced by the negative influence of 5-HT depletion on mood, the antidepressant efficacy of SSRIs, the perturbed 5-HT turnover and neuronal sensitivity in MDD patients and animal models, and the numerous associations between 5-HT pathway gene polymorphisms and MDD (**Table 1**).

**Table 1:** Serotonergic gene polymorphisms in MDD.

Reference	Candidate gene	Sample size	Main findings
(40)	serotonin transporter (SERT)	30 (15 healthy controls)	Compared to controls, MDD patents showed reduced SERT in brain.
(41)	5-hydroxyindoleacetic acid (5-HIAA)	68 depressed subjects	Lower 5-HIAA predicted suicide attempt in MDD.
(42)	5-HIAA, SERT	10 matched pairs	5-HIAA and SERT deficiency in depression.
(43)	serotonin transporter (5-HTT) and the serotonin-transporter-linked polymorphic region (5-HTTLPR)	220 subjects	Lower 5-HTT binding related to suicide and MDD. 5-HTTLPR related to MDD but not to suicide or 5-HTT binding.
(11)	5-HTT	1,037 subjects	Short allele of the 5-HTT promoter related to depressive symptoms, diagnosable depression, suicide, and stressful life events.
(44)	5-HTT	549 twins	Individuals expressing 2 short (S) alleles most sensitive to the depressogenic effects of stressful life events.
(45)	The intron 2 (STin2) polymorphism of the serotonin transporter	258 (152 controls)	The STin2 variant predicts suicide in MDD.
(46)	STin2 polymorphism of the serotonin transporter	170 (99 healthy controls)	Significant difference in the genotype frequency of STin2.10/10 in MDD.
(47)	5-HTT	66 (43 healthy controls)	Lower 5-HTT binding potential proportional to the number of available transporters in individuals with childhood abuse.
(48)	the serotonin transporter gene (SLC6A4)	98 subjects	Depressed mood during the 2nd trimester of pregnancy negatively correlated with maternal SLC6A4 promoter methylation status.
(49)	SLC6A4	108 depressed subjects	SLC6A4 methylation status related to childhood adversities and MDD.
(50)	SLC6A4	84 twins	Serotonin transporter receptor gene methylation variation in peripheral blood leukocytes positively related to depressive symptom severity.
(51)	SLC6A4	100 (50 healthy controls)	Compared with healthy controls, no significantly differed with MDD.
(52)	SLC6A4	94 depressed subjects	Reduced SLC6A4 expression related to impaired antidepressant treatment response after 6 weeks.
(53)	SLC6A4, and Serotonin 2A receptor (5-HT_2A_R)	137 depressed subjects	SLC6A4 AA genotype and A-allele related to antidepressant response.
(54)	SLC6A4	43 (24 healthy controls)	No significant associations with MDD.
(55)	SLC6A4	36 depressed subjects	Three candidate genes, including SLC6A4 related to the etiology of MDD and suicide attempts in Chinese.
(56)	SLC6A4	224 (150 healthy controls)	SLC6A4 allelic variations related to suicidal ideation in MDD.
(57)	SLC6A4	370 Parkinson's Disease patients	SS genotype predicts higher depression risk in Parkinson's disease.
(58)	5-HTTLPR	150 depressed subjects	No significant associations with MDD.
(59)	5-HTTLPR	136 (68 healthy controls)	SS genotype and S allele of 5-HTTLPR related to MDD in children.
(60)	5-HTTLPR	1,206 twins	No association between 5-HTTLPR and MDD.
(61)	5-HTTLPR	316 (125 healthy controls)	L^G^ and S allele positively correlated with MDD in patients experiencing moderate to severe life events.
(62)	5-HTTLPR	4,175 depressed subjects	Significant association between social adversity and MDD prevalence.
(63)	5-HTTLPR	306 males	The 34-item Childhood Trauma Questionnaire (CTQ) score and 5-HTTLPR level are independent risk factors predicting suicide attempt.
(64)	5-HTTLPR	233 depressed subjects	Associations among 5-HTTLPR polymorphisms, comorbid disorders, and sex in MDD.
(65)	5-HTTLPR	103 depressed subjects	5-HTTLPR SS genotype related to poor antidepressant response in females.
(66)	5-HTTLPR	984 subjects	Trauma was a risk factor for depressive symptoms who carries S/S or S/L genotype.
(67)	5-HTTLPR and Serotonin 2A receptor (5-HT_2A_R)	132 depressed subjects	5-HT_2A_ A-allele associated with MDD, 5-HTTLPR S allele associated with higher irritability score.
(68)	5-HTTLPR	104 depressed subjects	Statistical association between MDD and 5-HTTLPR L allele.
(69)	5-HTTLPR	121 (66 healthy controls)	No significant associations with MDD.
(70)	5-HTTLPR	1,111 subjects	Limited role of 5-HTTLPR in mediating effects of adolescent/parent relationship on depressive symptoms.
(71)	5-HTTLPR	73 (18 healthy controls)	Decreased fractional anisotropy (FA) related to 5-HTTLPR-S′L′in MDD.
(72)	5-HTTLPR	57 (29 healthy controls)	5-HTTLPR genotype related to mean methylation levels in MDD.
(73)	5-HTTLPR	160 depressed subjects	5-HTTLPR polymorphisms related to dysphoria score on Montgomery-Åsberg Depression Rating Scale (MADRS).
(74)	5-HTTLPR	178 depressed subjects	5-HTTLPR genotype predictive of resistance to SSRI treatment.
(75)	Serotonin 2A receptor (5-HT_2A_R) and 5-HTTLPR	136 (69 healthy controls)	5-HT_2A_ promoter -1438A variant associated with depressive symptoms of seasonal affective disorder.
(76)	Serotonin 1A receptor (5-HT_1A_R)	263 (134 healthy controls)	Compared to the healthy controls, depressed individuals twice as likely to carry -1019G genotype.
(77)	5-HT_2A_R	251 (131 healthy controls)	5-HT_2A_R 102C allele significantly associated with MDD, particularly in patients with suicidal ideation.
(78)	5-HT_1A_R	24 (8 healthy controls)	Decreased 5-HT_1A_R binding potential in MDD compared to controls.
(79)	HTR1A, HTR2A, HTR6, TPH1 and TPH2	481 (395 healthy controls)	No significant associations with MDD.
(80)	5-HT_2A_R	56 depressed subjects	AA genotype of 5-HT_2A_R -1438 G/A polymorphism related to sexual dysfunction in male MDD patients.
(81)	5-HT_2A_R and Serotonin 3A receptor (5-HT_3A_R)	50 (25 healthy controls)	Increased 5-HT_2A_R mRNA expression in peripheral blood mononuclear cells of MDD patients.
(82)	SERT, 5-HT_1A_R, and 5-HT_2A_R	167 depressed subjects	Lower SERT binding associated with MDD. Both greater 5-HT_1A_ binding and 5-HT_2A_ binding associated with MDD.
(33)	5-HT_1A_R	25 depressed subjects	Reduced 5-HT_1A_R binding potential in MDD.
(83)	5-HT_1A_R, 5-HT_2A_R and SERT	76 brain samples	Lower 5-HT_2A_ receptor binding in Brodmann areas 41/42 of MDD patients.
(84)	HTR_1A_	800 (400 healthy controls)	5-HTR_1A_ C (−1,019) G polymorphism significantly related to MDD.
(85)	HTR_2A_	1,282 (325 MDD patients, 155 BP patients and 802 healthy controls)	No significant associations.
(86)	HTR_1A_	1,135 (804 healthy controls)	No significant associations.
(87)	HTR_1A_, HTR_2A_	2,023 depressed subjects	No significantly associated SNP at genome-wide level.
(88)	HTR_1A_	81 (62 healthy controls)	HTR_1A_ rs6295 genotype related to MDD.
(89)	Tryptophan hydroxylase-2 (TPH2) and 5-HT_2A_	564 (287 healthy controls)	TPH2/5-HT_2A_ interaction influences MDD susceptibility.
(90)	Serotonin 4 (5-HT_4_) receptor	96 (48 depressed subjects, 48 schizophrenia subjects)	Associations between HTR4 polymorphisms and mood disorder.
(91)	5-HT_4_	57 healthy subjects, including 26 subjects had a family history of MDD	Association between the family history of MDD and lower striatal 5-HT_4_ receptor binding.

Changes in 5-HT signaling may also predict suicidality. Patients with suicidal impulses exhibited lower cerebrospinal fluid (CSF) concentrations of the 5-HT metabolite 5-hydroxyindoleacetic acid (5-HIIA) and fewer 5-HT uptake sites on platelets (92, 93). Weissmann et al. reported increased editing of the 5-HT_2C_ receptor (5-HT_2C_R) mRNA in cortical areas of depressed suicides compared to non-psychiatric controls, suggesting that region-specific changes in 5-HT_2C_R function may contribute to MDD etiology (94). Further, altered activities of the major 5-HT biosynthetic enzymes tryptophan hydroxylase 1 and 2 (TPH 1 and TPH 2) (95), of 5-HTT (96), and of serotonin receptors, especially HTR_1A_ (97), HTR_2A_ (98), and HTR_2C_ (99), are associated with suicidal impulses and violent suicidal behavior. However, contradictory findings have been reported (98, 100, 101), possibly due to low statistical power or heterogeneity of study populations. Larger-scale studies of different clinical and ethnic populations may resolve these controversies.

In animal models, genetic and pharmacological manipulation of serotonergic signaling can induce acute depression- and anxiety-like behaviors (102). Further, manipulating serotonergic and dopaminergic signaling during development can affect later-life somatosensory, anxiety/depression-like, and aggressive behavior (103). A recent study found generally lower levels of all three monoamines in a Wistar–Kyoto (WKY) animal model of maternal depression compared to matched control Sprague–Dawley (SD) rats (104).

Norepinephrine (NE) secreted from the locus coeruleus (LC) is a critical modulator of neural circuits involved in learning and memory (105–107), mood, sleep, appetite, and neuroendocrine function (108). Moreover, the antidepressant actions of monoamine oxidase (MAO) inhibitors and non-selective monoamine reuptake blockers suggest that NE plays a major role in the neurobiology of MDD (109). One potential pathogenic mechanism is elevated NE sensitivity of α_2_-adrenoceptors, which can inhibit NE release from the LC *via* negative feedback (110, 111). Indeed, elevated density and enhanced activity of α_2_-adrenoceptors have been reported in the brain tissues and platelets of MDD patients (112, 113). Elevated α_2_-adrenoceptor density has also been found in the frontal cortex and hippocampus of depressed suicides (114, 115). Moreover, Rivero and co-workers found that the elevated α_2_-adrenoceptors density in the prefrontal cortex of suicidal depressed subjects was resistant to antidepressant therapy, whereas elevated β_1_-adrenoceptor density was reduced by such therapy (116).

The efficacy of selective norepinephrine reuptake inhibitors (SNRIs) provides the strongest evidence for a direct contribution of deficient NE transmission to depression. A recent systematic review concluded that the SNRI duloxetine hydrochloride was effective against MDD as well as panic disorder, obsessive–compulsive disorder, and other psychiatric disorders (117), indicating broad involvement of NE in psychopathology. Another review suggested that duloxetine may be safe for older adults with MDD (118), although this agent has not been suggested for use as first-line acute therapy for MDD (119). Nonetheless, the norepinephrine transporter (NET) is well documented therapeutic target for MDD and like SSRIs (120), nonselective 5-HT/NE reuptake inhibitors such as venlafaxine (121) are widely used for MDD treatment. Many studies have also implicated NET gene polymorphism in MDD pathogenesis (**Table 2**). Abnormalities of noradrenergic function may also be involved in the pathogenesis of suicide (148). Several earlier studies reported upregulation of β-adrenoceptors in the brains of suicides (114, 149, 150), although several others reported the opposite (150, 151). Aside for receptor abnormalities, excessive stress could trigger depletion of NE and the onset of MDD (152).

**Table 2:** Dopaminergic and noradrenergic gene polymorphisms in MDD.

Reference	Candidate gene	Population/sample size	Main findings
(122)	Norepinephrine transporter (NET)	34 brain tissue samples (19 healthy controls)	Reduced NET in the LC related to MDD.
(123)	NET	179 (74 healthy controls)	No significant associations.
(124)	NET	200 (100 healthy controls)	No significant associations.
(125)	NET	248 (136 healthy controls)	Tendency for lower TT genotype frequency in MDD.
(126)	NET and 5-HTT	96 depressed subjects	T-allele of NET T-182C polymorphism associated with better antidepressant response.
(127)	NET	309 (164 healthy controls)	C/C genotype related to low MDD risk.
(128)	NET	426 (210 healthy controls)	No significant difference.
(129)	NET	776 (388 healthy controls)	Selected NET gene polymorphisms influence MDD risk from negative life events.
(130)	NET and 5- HTTLPR	579 depressed subjects	Both NET and 5-HTTLPR related to MDD, while the interaction between them associated with depression and Hamilton Depression Rating Scale for Depression baseline scores.
(131)	NET, and 5-HTTLPR	252 depressed subjects	No significant associations between selected polymorphisms and antidepressant response.
(132)	the norepinephrine transporter (SLC6A2), HTR_1A_, and COMT	126 depressed subjects	No significant associations between SLC6A2 polymorphisms and antidepressant treatment response.
(133)	SLC6A2, TPH2	205 depressed subjects	SLC6A2 polymorphism related to MADRS-defined olanzapine+fluoxetine response in MDD.
(134)	SLC6A2	550 (201 with MDD and suicide attempts, 160 with MDD without suicide attempts, and 189 healthy controls)	SLC6A2 polymorphism related to suicide risk in MDD.
(135)	NET	604 (302 healthy controls)	CC genotype of NET gene may reduce risk of depression.
(136)	SLC6A2	243 depressed subjects	Association between SLC6A2 gene variation and remission after venlafaxine treatment in MDD.
(137)	NET	776 (388 healthy controls)	Significant association between T-182C polymorphism and MDD.
(138)	SLC6A4, NET, HTR_1A_, HTR_2A_, COMT, and brain-derived neurotrophic factor (BDNF)	53 (27 healthy controls)	No difference in NET polymorphisms between MDD group and controls.
(139)	NET	78 (48 healthy controls)	Significant diagnosis interaction for NET G1287A polymorphism in MDD.
(140)	DRD4, TPH, MAO-A, and 5-HTTLPR	134 nuclear families with mood disorders (58 with MDD)	No significant associations.
(141)	DRD4, MAO-A, 5-HTTLPR, DRD2, and DAT1	United States	DRD4 5-repeat allele related to depressive symptoms among adolescents/young adults.
(142)	DAT1	264 depressed subjects	DAT1 VNTR polymorphism related to antidepressant response.
(143)	DAT1	Russia	DAT1 polymorphism rs40184 related to MDD and suicidal ideation.
(144)	DAT1	Chinese	No significant associations.
(145)	DAT1, COMT	German	9R/9R and Val/Val genotype negatively related to Sadness score.
(146)	DAT1	1,714 subjects	DAT1 related to children's depressive symptoms.
(147)	DAT1 and COMT	Chinese	Interaction of DAT1, COMT, and peer acceptance predictive of adolescent depressive symptoms.

While 5-HT and NE are the biogenic amines most consistently associated with MDD, abnormalities in DA signaling have also been implicated. For instance, depletion of DA has also been reported in MDD patients (153). The medial part of the VTA projects mainly to the nucleus accumbens and ventral striatum, which are central hubs of the brain reward system (154, 155). Allelic variation of DA-related genes modulate brain circuitry involved in the regulation of negative emotional stimuli (156), and DA system dysfunction has been associated with many symptoms of MDD such as anhedonia and low motivation (157, 158), as well as with cognitive symptoms such as impaired concentration (159, 160).

A dopamine deficiency has also been reported in MDD. One study measuring monoamine neurotransmitters and related metabolites in the cortex of rats detected DA only in the control group (161). A multi-data source-based prioritization (MDSP) study by Liu et al. identified 143 depression-related genes, including the DA receptor 4 (DRD4), as well 16 significantly enriched Kyoto Encyclopedia of Genes and Genomes (KEGG) pathways, including the ‘dopaminergic synapse' as well as the ‘serotonergic synapse' and ‘glutamatergic synapse'. The neuroactive ligand–receptor interaction list from KEGG pathway analysis also included the dopaminergic synapse (162). Further, a number of dopaminergic gene polymorphisms are associated with MDD (**Table 2**).

Reduced NE, 5-HT, and DA have been identified as significant biomarkers for depression in animal studies (163, 164). Advances in imaging techniques, including PET and single-photon emission computed tomography (SPECT), have also provided valuable insights into the contributions of DA to MDD. For instance, a recent study reported significantly reduced DA transporter (DAT) availability in the bilateral putamen and VTA of patients compared to healthy controls (Cohen d range, −0.62 to −0.71) (158). Moreover, this same study found lowest DAT availability in the VTA of patients reporting the greatest stress-related fatigue (165). While this relationship was replicated (166), the findings of a meta-analysis were contradictory (167).

In summary, the evidence is very strong that dysregulation of NE, DA, and 5-HT signaling contributes to MDD development and symptom expression. However, prospective studies are required to establish causal relationships between these deficiencies and MDD.

## Personality Traits and MDD

Personality can be described as a composite of multiple, relatively stable traits and specific trait profiles, as measured using instruments such as the Neuroticism, Extraversion, Openness Five-Factor Inventory (NEO-FFI) questionnaire, Temperament and Character Inventory (TCI), and Eysenck Personality Questionnaire (EPQ) for associations with MDD risk.

A large-scale longitudinal cohort study using baseline and 2-year follow-up data found that increased neuroticism scores on the NEO-FFI were associated with both anxiety and depressive disorders. Higher agreeableness has also been associated with the occurrence of MDD, while openness demonstrated no association with the occurrence of, or recovery from, any depressive or anxiety disorder (168). In contrast, extraversion trait scores were associated with lower depressive disorder incidence and increased rate of recovery (169). Pair-wise genome-wide association studies (GWASs) have also found that numerous genetic variants overlap between depression and trait neuroticism (170). Further, high trait neuroticism has been confirmed as a dominant risk factor for depression (104). Also, low extraversion scores were a predictor of depression during the remission period of bipolar disorder (BP), the other main subtype of mood disorder (171). A recent resting-state dynamic functional network connectivity analysis found that state 4 was positively correlated with trait extraversion and negatively correlated with neuroticism, as measured by the EPQ, and that MDD patients showed significantly reduced dwell time and fractional time in state 4 compared to healthy controls, with lowest centrality degree in hippocampus and ventral striatum (172).

Neuroticism can improve the ability to cope with negative emotional stimuli (173) and has been linked to panic disorder (174), schizophrenia (175), and obsessive–compulsive disorder (OCD) (176) as well as to MDD. According to twin studies, the heritability of trait neuroticism is approximately 40%, with 15% to 37% caused by single-nucleotide polymorphism (SNP) variations (177). High trait neuroticism is associated with sensitivity to stress and negative emotional experiences, as well as with excessive worry, emotional vulnerability, and increased emotional exhaustion (178), all of which can impact an individual's physical activity (179), perception (180, 181), and emotion (182). An early meta-analysis of GWASs analyzing over 106,000 individuals identified nine neuroticism-associated loci (including the ionotropic kainate 3 glutamate receptor, Kelch-like protein 2, and corticotropin-releasing hormone receptor 1). This same study also found a strong association between neuroticism and MDD (genetic correlation = 0.64), but no sex difference in the heredity of neuroticism (177). Another meta-analysis of GWASs identified the Membrane-associated guanylate kinase inverted repeat member 1 (MAGI1) gene as a novel locus for neuroticism, both among the entire cohort of 63,661 individuals as well as in the combined Netherlands Twin Registry (NTR)/Netherlands Study of Depression and Anxiety (NESDA) cohort, with significant polygenic risk scores associated with MDD for SNP sets at P-value thresholds of 0.01 and 0.05, again providing compelling evidence that higher neuroticism is strongly correlated with MDD (183).

Harm avoidance (HA), a core personality trait defined by Cloninger, reflects a tendency to avoid potential danger, and like neuroticism, is related to traits such as pessimism, anxiousness, insecurity, bashfulness, and unusual susceptibility to fatigue (184). Trait HA has a high degree of stability throughout life (185), and is strongly associated with OCD (186), eating disorders (187), and other psychiatric disorders. High HA scores are also considered predictive of MDD (188). Bipolar disease patients demonstrating high HA scores on the TCI also showed a strong tendency for poor antidepressant treatment response during depressive episodes (189). A meta-analysis focusing on the associations between personality traits and MDD recovery found that patients with high novelty seeking (NS), high self-directedness (SD), and low HA exhibited better antidepressant responses (190). Alternatively, higher HA scores and lower SD scores were significantly correlated with non-remission in MDD patients (191), these findings have been replicated (192–194). Interestingly, a meta-analysis from Zaninotto et al. not only found such correlations, but the team reported the influence of HA in MDD vs healthy subjects was significantly greater than that found in BP vs healthy subjects (195), although there was marked heterogeneity among the included studies. Additional longitudinal studies are needed to confirm the association between HA and MDD.

Personality traits are also the major focus of suicide research. Garcia Herrero et al. concluded that high neuroticism can predict suicidal ideation (196). Similarly, Peters and his colleagues followed a large sample population in the United Kingdom for 10 years and found that neuroticism was related to suicide risk in both males and females and that neuroticism was a major predictor of suicide in females with mood disorders (197). An earlier study also found that neuroticism and openness were risk factors for suicide specifically in females, while extraversion and conscientiousness reduced the risk in males (198).

A recent study using the TCI to assess personality traits found that higher HA increased the risk of suicidal ideation in depression (199). Eric et al. also reported significantly higher HA scores, as well as low SD scores in subjects with suicidal ideation (192). Further, several studies have found that higher HA and NS scores are significant risk factors for suicidal behavior (200–202), while others have linked lower SD and higher self-transcendence (ST) to suicidality (203, 204).

Mood state may also impact personality traits, at least as measured at specific times, which complicates these association results. Nonetheless, the relatively consistent relationships between specific traits and MDD, including suicidal MDD, and the overlap between several trait-related and MDD-related genes suggest that investigations of the genetic and physiological attributes underlying specific traits may provide additional clues to the pathophysiology of MDD.

## Monoamine Neurotransmitters and Personality Traits

Twin, family, and genomic studies have shown that personality traits are strongly influenced genetics, with estimated heritability ranging from 40% to 60% (205–208). Cloninger's Tridimensional Personality Questionnaire (TPQ) traits NS, HA, and reward-dependence (RD) have all been associated with monoamine functions (209, 210), as have the so called “the Big Five” personality traits assessed by NEO, NEO-PI-R, and NEO-FFI (neuroticism, extraversion, openness to experience, agreeableness, and conscientiousness) (211) and the three personality traits of the EPQ (psychoticism, extraversion, and neuroticism) (212).

Extraversion, a higher-order personality trait, has been linked to reward system function in several studies (213–215). Furthermore, evidence strongly suggests that DA modulation is involved in both reward system function and extroversion (216). Smillie et al. (208) and co-workers reported that subjects with the DA receptor 2 (DRD2) gene A1-allele had significantly higher extroversion scores. In contrast, however, a functional magnetic resonance imaging (fMRI) study reported that A1-allele carriers exhibited lower extraversion scores, although the difference between carriers and non-carriers was not significant (217). A cross-national study of personality differences by Fischer et al. found a positive correlation between dopaminergic brain function index score and extraversion as well as a negative association between dopaminergic function and neuroticism score in those under high stress (218). A meta-analysis also found a relationship between self-consciousness (one facet of neuroticism) and the domain receptor 1 (DDR1) gene (219). Again, these relationships may be complicated by covariables. For instance, a previous study reported a negative correlation between neuroticism scores and quality of life in schizophrenia (175).

The opponent interactions between serotonin and DA makes the relationship between serotonin and personality traits was interesting and complex (220). Several studies have looked at the relationship, but the results have been inconsistent (**Table 3**). For example, most evidence to date support a link between the serotonin-transporter-linked polymorphic region (5-HTTLPR) and neuroticism (252, 286), meanwhile the different result were obtained using NEO-FFI (225). Interesting, in Swedish cohort study, they observed openness was significantly associated with 5-HTTLPR, while they also found that the positive association between openness and childhood adversity in the gene-environment model regardless of 5-HTTLPR genotype (225). Paaver et al. demonstrated S allele carriers with adverse family relations were related to higher thoughtlessness, disinhibition and impulsivity using the Barratt Impulsiveness Scale 11 (BIS-11) solely among girls (254), they also indicated that, in agreement with other studies, the influence of 5-HTTLPR genotype on affect is related to environmental adversity (61, 66). Indeed, environmental adversity, such as childhood adversity, can have a negative effect on child's expectations and present strained interpersonal relationships, which can affect personality or temperature (295), as well as associate with a range of psychopathology, including MDD (11). This factor has not been considered in some studies, which might be one of the fundamental reasons for the inconsistent results. Some studies on children have demonstrated significant association between 5-HTTLPR short (S) allele and higher NS scores (253), and S allele closely related to higher prevalence of substance use (296). In addition, the study of the relationship between personality trait and NE is rather little.

**Table 3:** Relationships between monoaminergic system function and personality traits.

Reference	Sample size	Approach	Main findings
(221)	290 (147 males and 143 females)	Zuckerman–Kuhlman–Aluja Personality Questionnaire	Four tagged single-nucleotide polymorphisms (tagSNPs), including DRD4, were related to Neuroticism and the 4 tagSNPs, including DRD2 and DRD4, were associated with Sensation Seeking.
(222)	99 females	NEO	MAOA-u variable number of tandem repeats (VNTR) polymorphism significantly associated with trait Neuroticism. No associations with COMT Val^158^ Met, 5-HTTLPR, or DAT 3'UTR VNTR.
(223)	600 males	NEO-FFI	DRD4 significantly related to extraversion, the DAT1 to agreeability.
(218)	127,685 subjects	NEO-PI-R and Occupational Personality Questionnaire (OPQ)	Dopamine-system only in climatic stress closely related to personality trait Neuroticism and Extraversion. Interaction between dopamine and climatic demands significant for Openness/Intellect on OPQ scores.
(224)	50 males	TCI	5-HT_1A_ receptor binding not associated with ST/SA scores.
(225)	3,112 subjects	Swedish translation of Schafer's FFM rating scale	Openness (to experience) associated with serotonin-transporter-linked polymorphic region.
(226)	1,139 (550 males and 589 females)	Short-form Maudsley Personality Inventory (MPI)	Serotonin transporter polymorphisms (5-HTTLPR and rs25531) associated with Neuroticism in males.
(227)	69 (51 males and 18 females)	NEO	No association between personality traits and 5-HT_4_R.
(228)	147 (91 males and 56 females)	NEO-PI-R NEO-FFI,	Neuroticism positively associated with serotonin transporter binding potential in males, negatively associated with serotonin transporter in females.
(229)	44 (22 males and 22 females)	Karolinksa Scales of Personality	Explicit associations between the D2/3R and the trait impulsivity.
(230)	61 (47 males and 14 females)	Buss–Perry Aggression Questionnaire (BPAQ) and Barratt Impulsiveness Scale	Positive correlations of 5-HT_4_R with BPAQ total score and BPAQ physical aggression score in males.
(231)	272 females	NEO-FFI	Statistically significant relationship between Openness to experience score and the 5-HTT polymorphism. No significant relationship between NEO-FFI score and MAO-A polymorphism.
(232)	1,576 (675 males and 901 females)	Estonian version of Revised NEO Personality Inventory (NEO-PI-R)	Lower Neuroticism and higher Conscientiousness scores significantly related to tryptophan hydroxylase 2 (TPH2).
(233)	616 (273 males and 373 females)	NEO-FFI	Higher COMT enzymatic activity (GG) related to lower Neuroticism, higher Agreeableness, and higher Conscientiousness scores.
(234)	34 (18 males and 16 females)	Karolinska Scales of Personality	Negative relation between Neuroticism and serotonin 5-HT_1A_ receptor binding.
(235)	16 subjects	TCI	Self-transcendence was associated with serotonin transporter (SERT) availability.
(236)	575 (274 males and 301 females)	TPQ	HA2, HA3 and RD1 scores significantly associated with NTR1 polymorphism rs6090453. HA2 and total RD scores significantly associated with rs6011914. No associations between NS and the selected SNPs.
(237)	12 males	TPQ	Significant correlation between DA synthesis ability in the ventral striatum and NS3.
(238)	46 subjects	Eysenck Personality Questionnaire (EPQ-R)	No significant result.
(239)	599 (341 males and 258 females)	Zuckerman Kuhlman Personality Questionnaire (ZKPQ)	D4R promoter polymorphisms not related to Sensation seeking.
(240)	372 males	TCI and Eysenck personality questionnaire	Significant associations between Sensation seeking and both 5-HTTLPR and 5-HT2CR.
(241)	72 (41 males and 31 females)	NEO PI-R	Openness to experience was related DRD2-mediated transmission.
(242)	2075 subjects	TCI	Positive correlation between 5-HTT BPND and SD score.
(243)	94 (60 males and 34 females)	Buss–Perry Aggression Questionnaire (AQ) and BIS-11	No associations between 5-HT_2A_R and AQ or BIS-11 total scores.
(244)	418 (104 males and 314 females)	the Formal Characteristics of Behaviour–Temperament Inventory	Significant association between DAT1 polymorphism and sensory sensitivity. Sex/DRD4 interaction impacts the same trait.
(245)	1,084 (407 males and 677 females)	TCI	No significant association between -141C Ins/Del polymorphism or the DRD2/ankyrin repeat and kinase domain containing 1 (ANKK1) Taq1 A polymorphism and personality traits, but an ANKK1 × DRD2 interaction affects TCI scores.
(246)	502 (240 males and 262 females)	TPQ	No significant association between CK1ϵ and TPQ scores.
(247)	1,091 subjects	EPQ	No significant result.
(248)	20 males	NEO	Significant associations between low 5-HTT in the dorsal raphe nucleus and both straight forwardness and trusting personality.
(249)	21 (8 males and 13 females)	TCI	HA score negatively correlated with D2/3 receptor availability.
(250)	652 (222 males and 430 females)	Eysenck Personality Inventory (EPI) and Temperament and Character Inventory-125 (TCI-125).	Significant effects of ANKK1/DRD2 Taq1A on Neuroticism and of dopamine transporter gene (SLC6A3) rs27072 on Persistence in both sexes. Significant association between ANKK1/DRD2 Taq1A A2/A2-genotype and higher NS and lower RD in males. Significant association between SLC6A3 10R*G-haplotype and higher Persistence in females.
(251)	289 (123 males and 166 females)	TCI	No significant associations with TCI scores.
(252)	94 (14 males and 80 females)	Dutch personality questionnaire (DPQ)	5-HTTLPR S-allele increases affective reactivity to examination stress independent of trait Neuroticism.
(253)	216 (129 males and 87 females)	TPQ and Buss–Durkee Hostility Inventory	S allele of 5-HTTLPR was related to higher NS scores.
(254)	483 (222 males and 261 females)	BIS-11 and Adaptive and Maladaptive Impulsivity Scale	S allele of 5-HTTLPR was associated with high maladaptive impulsivity.
(255)	502 (240 males and 262 females)	TPQ	Significant associations between rs12601930C/T and the trait NS. Both rs879606A/G and rs3764352A/G associated with HA.
(256)	16 (8 males and 8 females)	Swedish universities Scales of Personality	Social desirability negatively correlated with D2-receptor availability in striatum.
(257)	21 (10 males and 11 females)	TCI	The different regions of 5-HT_2A_ affects Persistence independent of sex.
(258)	50 (35 males and 15 females)	NEO PI-R	Negative correlation between Openness to Experience and *in vivo* cerebral 5-HTT binding.
(259)	1,114 subjects	TCI	DRD2 related to Novelty seeking in childhood.
(260)	83 (52 males and 31 females)	NEO PI-R	Positive correlation between 5-HT_2A_ binding and Neuroticism.
(261)	549 (304 males and 245 females)	TCI	Monoamine oxidase A (MAOA-VNTR) gene high-activity allele exhibited significant higher P scores than low-activity gene in females.
(262)	301 subjects	EPQ and TCI	5-HTT gene S Tin2.10 allele associated with Neuroticism and HA.
(263)	31 subjects	NEO	Positive correlation between neuroticism and 5-HTT binding in the thalamus.
(264)	42 (19 males and 23 females)	Maudsley personality inventory	Lie scale related to striatal dopamine D2/D3 receptor availability.
(265)	324 subjects	TCI	Significant associations between monoamine oxidase A polymorphism and both NS and RD.
(266)	256 subjects	NEO PI-R	No significant interaction among three functional polymorphisms in the tyrosine hydroxylase, monoamine oxidase A, and COMT genes on personality traits.
(267)	370 females	TPQ	MAOA-uVNTR gene related to HA of TPQ, and the HA4 got the highest score.
(268)	149 (65 males and 84 females)	TCI and NEO-PI-R	Association between rs1050450 polymorphism and Openness on NEO. No association was found using TCI.
(269)	219 females	TCI	No significant associations between monoamine oxidase A promoter polymorphism and personality traits.
(270)	33 subjects	Karolinska Scales of Personality	High scores on somatic anxiety and muscular tension and irritability significantly associated with reduced [^18^F] fluorodopa uptake in the caudate.
(271)	15 males	TCI	5-HT_1A_ receptor binding potential (BPND) negatively correlated with ST/SA.
(272)	115 subjects	NEO-FFI	DRD4 exon III and -521C/T not related to any personality trait.
(273)	101 females	TCI	Association between DRD4 variants of DRD4 and both NS and P personality traits.
(274)	149 (57 males and 92 females), and 252 (103 males and 149 females)	TPQ	COMT gene polymorphism related to higher HA scores in females, with Met158/Met158 genotype most strongly associated.
(275)	66 males	TPQ and EPQ	EPQ correlated with [^11^C]WAY-100635 binding of 5-HT_1A_ receptors.
(276)	71 (33 males and 38 females)	NEO-FFI	Significant interaction of sex and DRD4 polymorphisms (-616 and -521C) related to Extraversion scores.
(277)	11 (8 males and 3 females)	TPQ	Cerebral cortex 5-HT_2A_ receptors associated with HA.
(278)	371 (206 males and 165 females)	Karolinska Scales of Personality, Scandinavian Universities Scales of Personality, Health-Relevant 5-Factor Personality inventory, TCI and NEO-PI-R	No association between MAOA promoter region and personality traits in Swedish population.
(279)	16 males	TCI	Significant relation between dopamine D2 receptor (D2R) and personality trait of HA.
(280)	24 males	TCI	NS scores negatively correlated with D2R.
(281)	19 (11 males and 8 females)	NEO-PI-R	Negative correlation between Neuroticism and cortical 5-HT_1A_ receptor.
(282)	577 subjects	TPQ	COMT and 5-HTTLPR significantly related to RD2 scores by grouping.
(283)	18 (10 males and 8 females)	Karolinska Scales of Personality	Negative correlation between dopamine transporter and detachment personality scores, especially in the right hemisphere.
(284)	86 subjects	TCI	DRD4 exon III -521C/T polymorphism significantly associated with NS, with higher scores for C/C genotype.
(285)	256 subjects	NEO PI-R	No association between extraversion and DRD4 polymorphisms.
(286)	902 (505 males and 397 females)	NEO-PI-R	Higher NEO Neuroticism related to 5-HTTLPR polymorphism.
(287)	69 females	TCI	Significant association between DRD4 exon III long allele and NS scores.
(288)	119 males	TPQ	Young males with all three minor DRD2 alleles and the DRD4 7R allele show the most significant difference in NS scores.
(289)	341 (204 males and 137 females)	TPQ	No significant difference between D4 dopamine-receptor (DRD4) and the trait NS.
(290)	126 subjects	Karolinska Scales of Personality	DRD4 polymorphisms not related to personality traits.
(291)	153 females	TCI	Dopamine D4 receptor (D4DR) polymorphic exon III related to NS subscale of Exploratory Excitability.
(292)	124 subjects	TPQ	Association between NS scores and D4DR polymorphisms.
(293)	115 subjects	TCI	Norepinephrine transporter T-182C gene polymorphism was associated with personality trait RD in Koreans.
(294)	270 subjects (117 males and 153 females)	NEO-FFI	NET gene polymorphisms related to extraversion.

A number of monoaminergic transmitter-related genes are linked to personality traits, such as those encoding catechol-O-methyl-transferase (COMT) (297), monoamine oxidase A (MAOA) (222), and glutathione peroxidase 1 (GP × 1) (268). Furthermore, polymorphisms in monoamine receptors, for example 5-HTTLPR(226) and DRD4 (221), are associated with personality traits (**Table 3**). Recent studies in our laboratory have demonstrated associations between personality traits and Neurotensin receptor 1 (NTR1) (236), Dopamine- and cAMP-regulated phosphoprotein (DARPP-32) (255), and casein kinase 1ϵ (CK1ϵ) (246), all of which can affect monoaminergic signaling.

Undoubtedly, it is important that any assessment of the role of monoamines in personality traits should involve precise neural circuits associated with the relevant behavioral processes from the examples provided above (298). However, in many studies, there are some limitations, such as the small sample size with low statistical power, still need more participants to provide high quality evidence in further analysis.

## Conclusions

MDD, therapeutic strategy still remain unclear, is one of the most prevalent medical disorder which causes life-threatening conditions, like suicides tend and suicidal behaviors. Although the precise etiology is not known, several studies support the fact that MDD is the severe mental disease that involves disturbance of chemical neurotransmitters, psychosocial factors, genetic factors, personality traits and other formulations. In our study, numerous strong associations have been identified among monoamine signaling deficits, detrimental personality traits, and major depressive disorder, providing potential clues to disease pathogenesis. And through incredible advancements in medical technology, these independent and interactive dimensions may be promising targets for precision medicine. Suicide is a massive public problem in depressed patients, thus research regarding the prevention and intervenient countermeasures of suicide should be thoroughly investigated in the field of biogenic amines changes and personality traits. Moreover, such studies have identified potential biomarkers for MDD risk that could aid in the early identification of at-risk individuals (299). Clinical programs should focus on early identification and intervention for emotional problems and high-risk behaviors among children and adolescents. Notably, the evidences for the relationship between monoamines, MDD and personality traits are confused and contradictory. Small sample size (significantly drop the accuracy rate and lead bias), unified analyzing methods, differences in tissues, depressive phenotypes, ethnicities, and others may lead to these inconsistent data. These factors should be considered in future studies.

## Author Contributions

GZ planned and directed the paper, and XS wrote it.

## Funding

This project was supported by a grant from the Major Project of the Department of Science & Technology of Liaoning Province (2019JH8/10300019) and a grant from the Major Project of the Science and Technology Ministry in China (2017YFC0820200).

## Conflict of Interest

The authors declare that the research was conducted in the absence of any commercial or financial relationships that could be construed as a potential conflict of interest.
